# Investigation of the Clinical and Genetic Spectrum of PMM2-CDG: Insights from a Family with a Novel Variant and Previous Studies

**DOI:** 10.34172/aim.34187

**Published:** 2025-07-01

**Authors:** Parnian Alagha, Tara Akhtarkhavari, Ebrahim Shokouhian, Fatemeh Ghodratpour, Sanaz Arzhangi, Hossein Najmabadi, Kimia Kahrizi

**Affiliations:** ^1^Genetics Research Center, University of Social Welfare and Rehabilitation Sciences, Tehran, Iran

**Keywords:** Congenital disorder of glycosylation type 1A, Genotype-phenotype correlation, Novel variant, PMM2 gene, Novel variant

## Abstract

**Background:**

PMM2-CDG, also known as congenital disorder of glycosylation type 1a, is the most common N-linked glycosylation disorder, characterized by a wide range of neurological and multisystem manifestations. Understanding the genotype-phenotype correlations is essential for accurate diagnosis and patient management. This study aims to identify the genetic cause of PMM2-CDG in an Iranian family with multiple affected members, and to analyze the genetic and clinical spectrum of the disorder through a comprehensive literature review.

**Methods:**

Exome sequencing re-analysis was performed to detect disease-causing variants in three affected siblings. Additionally, a literature review was conducted, analyzing 91 previously reported cases of PMM2-CDG to determine the most prevalent variants and associated clinical features.

**Results:**

A novel splice site variant (c.640-9T>A) was identified alongside a previously reported missense mutation (c.647A>T; p.N216I) in the affected individuals. The literature review revealed that the most frequent *PMM2* variants were p.R141H (28.8%), p.V231M (12.8%), p.N216I (6.4%), and p.V129M (5.8%), with 77.6% of mutations occurring in exons 5 and 8. The most common clinical findings included developmental delay, ocular abnormalities (hypertelorism, strabismus), muscular system defects (hypotonia, muscle weakness), neurological symptoms (abnormal MRI findings), cardiovascular involvement (pericarditis, pericardial effusion), and clotting disorders.

**Conclusion:**

We expect that our detailed clinical study will improve the genotype-phenotype interpretation of causal PMM2-CDG variants and the analysis of next-generation sequencing data, leading to clarification of the cause of complicated cases of rare diseases.

## Introduction

 Congenital disorders of glycosylation (CDGs) constitute a heterogeneous group of inherited metabolic diseases characterized by defects in glycoprotein and glycolipid glycan synthesis and attachment. Over 160 CDG subtypes have been described,^1,2^ encompassing N-linked, O-linked, and hybrid N- and O-linked glycosylation, as well as lipid and glycosylphosphatidylinositol (GPI) anchor biosynthesis abnormalities.^3^ The first CDG was identified by Jaeken and colleagues in 1980, affecting approximately 1 in 20 000 individuals.^3-5^

 Clinically, patients often present with a recognizable phenotype characterized by neurological and multisystem manifestations, which can complicate the diagnosis of CDG. The severity of PMM2-CDG varies widely, ranging from severe neonatal forms with a high mortality rate (approximately 20% within the first year of life) to milder presentations in adulthood.^5-7^ Neurological signs are the primary clinical feature of PMM2-CDG, affecting both the central and peripheral nervous systems. These neurological abnormalities may occur alone or alongside systemic abnormalities.^2^

 During infancy, the affected individuals frequently present with neurological deficits, including cerebellar hypoplasia, hypotonia, ataxia, and hyporeflexia, as well as strabismus. Additionally, failure to thrive, hepatic problems, and developmental delay are commonly observed.^3,8^ Hypotonia, ataxia, retinitis pigmentosa, seizure, intellectual disability (IQ 40‒70), stroke-like episodes, speech and movement impairments, peripheral neuropathy, coagulopathy, and skeletal abnormalities are common features in the affected children.^3^ Retinitis pigmentosa, myopia, joint contractures, non-progressive cognitive dysfunction, and peripheral neuropathy are common clinical findings in adolescents with PMM2-CDG.^9^

 PMM2-CDG results from mutations in the *PMM2* gene, located on chromosome 16p13.2, which encodes a 246-amino acid protein. This gene is broadly expressed in both human and mouse tissues.^10^ As a member of the HAD-IIB phosphomutase subfamily within the larger HAD superfamily of hydrolases, PMM2 possesses a conserved alpha/beta core domain, a structural feature shared among homologs spanning bacteria, archaea, and eukaryotes.^11^ Structurally, PMMs are composed of a core domain (residues 1‒90 and 198‒262) that houses the active site with four conserved motifs, and a cap domain (residues 95‒194), which plays a role in enzyme function and stability.^10,12,13^

*PMM2 *encodes a homodimeric cytosolic isomerase that catalyzes the conversion of mannose-6-phosphate to mannose-1-phosphate in the cytosol, with glucose 1,6-bisphosphate serving as an activator.^3,5,14^ Mannose-1-phosphate is an essential precursor for synthesizing GDP-mannose and dolichol-phosphate-mannose, both of which serve as mannose donors in N-linked glycosylation pathways.^5,15,16^ N-linked glycosylation is an important post-translational modification that involves a variety of processes including protein folding, signaling, trafficking, protein stability, localization, cell adhesion, etc.^17,18^ In addition to its extensive role in cellular functions, the importance of *PMM2* is highlighted by a study demonstrating that targeted disruption of the *PMM2* gene in mice results in early embryonic lethality.^10,19^

 Enzymes responsible for catalyzing N-glycosylation are ubiquitously expressed throughout both developing and adult nervous tissue.^18^ Studies have demonstrated the vital role of N-glycosylation in both neurodevelopmental processes and the functioning of the mature brain.^20^ N-glycosylation is essential for neuronal function, influencing various cell types including neurons, astrocytes, and microglia.^20^ Furthermore, fucosylated glycans, synthesized from GDP-mannose,^21^ play a crucial role in cognitive processes such as learning and memory.^22^ These findings align with the clinical observation that almost all patients with PMM2-CDG exhibit neurological symptoms.

 Here, we describe an Iranian family with three individuals affected by the rare congenital disorder of glycosylation type 1a, who have a compound heterozygote variant in the* PMM2* gene; it is the third family with PMM2-CDG reported from Iran with a new nucleotide substitution. Our results further underscore the importance of a thorough and systematic re-evaluation of phenotypic descriptions, alongside using an up-to-date and reviewed pipeline for reanalysis of WES data.

## Materials and Methods

 An Iranian family (from Babol city, northern Iran) was previously referred to the Genetics Research Center (GRC) of the University of Social Welfare and Rehabilitation Sciences (USWR) for genetic investigation of intellectual disability, but the pathogenic variant(s) were not identified in our previous NGS investigation of the family. We performed a re-analysis study on the family, clinical re-examinations were conducted for affected individuals by a specialist clinician, and the clinical records were reviewed. Written informed consent was obtained from the parents of the patients and normal siblings. The study was approved by the Ethics Committee of the University of Social Welfare and Rehabilitation Sciences, Tehran, Iran. Peripheral blood samples were collected and genomic DNA was extracted using the salting-out protocol.

 Samples collected from the proband of the family underwent re-sequencing using the Agilent SureSelectXT Human All Exon V6 Kit (Agilent Technologies, Santa Clara, CA, USA), and sequencing was performed on Illumina NextSeq500 (Illumina, San Diego, CA, USA). Since the GATK platform was used in the previous analysis, in this re-analysis, in addition to aligning raw sequenced data against the human reference genome hg38/GRCh38, sorting, duplicate marking, base quality recalibration, and small variant SNV and indel calling were performed using the Illumina DRAGEN Bio-IT Platform V3. The generated VCF file was uploaded to Ilyome (https://www.ilyome.com) for re-annotation and re-analysis. Variant filtering on the Ilyome platform was conducted by considering their quality (depth greater than 3) and allele frequency (less than 1% in gnomAD genomes, gnomAD exomes, Genoks, 1000 genomes, and TOPMED bravo databases). In the next step of variant filtering, variants including stop gained, frameshift, stop or start lost, transcript amplification, in-frame insertion and deletion, missense, protein-altering, and splice region variants with good coverage would remain for analysis. Variants were prioritized based on variant impact, inheritance patterns, phenotype compatibility, allele count in population databases (allele count for homozygous and heterozygous was 0 and less than 10, respectively), and *in-silico* prediction scores. Additionally, the analysis of variants involved the utilization of various databases, including Online Mendelian Inheritance in Man (OMIM, https://www.omim.com), ClinVar (https://www.ncbi.nlm.nih.gov/clinvar), Varsome (https://varsome.com), Franklin (https://franklin.genoox.com), and PubMed, as well as *in-silico* prediction tools such as SIFT, MutationTaster, REVEL, MetaRNN, CADD, dbscSNV, and SpliceAI (for splicing variants).

 Furthermore, we conducted a comprehensive review of published studies reporting *PMM2* variants and associated clinical data from 2017 to 2024 as this is a descriptive study aimed at investigating additional genotype-phenotype correlations. PubMed and Google Scholar were used as primary databases for this review. We excluded papers that reported solely clinical data, exclusively molecular data, or provided cohort-level data without individual patient clinical information.

## Results

###  Family History and Clinical Presentation

**Figure 1:**
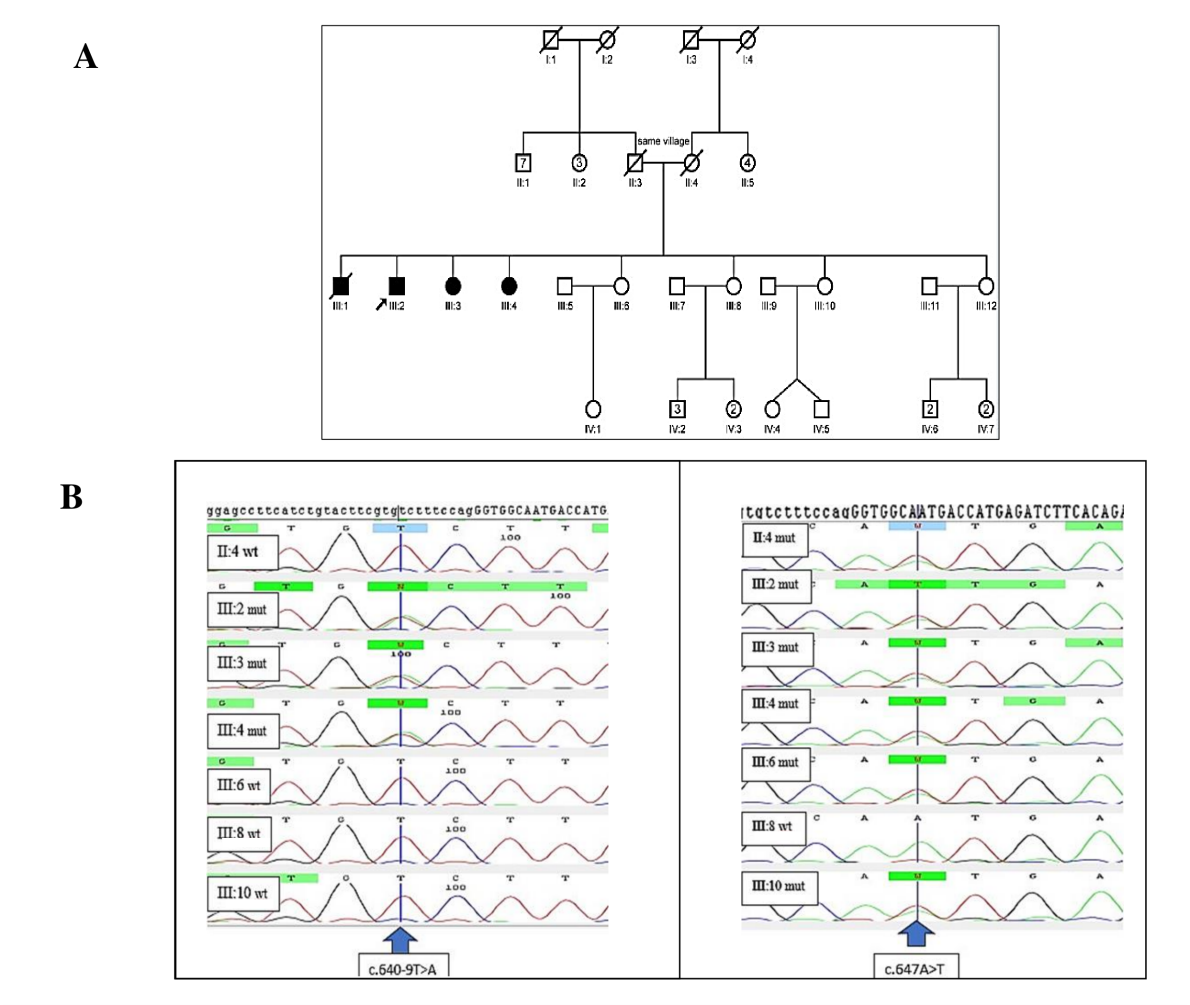
(A) Pedigree of the family. (B) Sanger sequencing of the c.647A > T and c.640-9T > A variants in the family. Wild type (wt) and mutated sequences; Arrow shows the mutated position

 The family had three affected siblings; two females (aged 61 and 46 years) and one male (aged 68 years), born to unrelated parents but originating from the same village. All presented with developmental delay, microcephaly, severe intellectual disability, strabismus, short stature (150 cm, 130 cm, and 145 cm, respectively), hypotonia, and spastic paraplegia. Hearing impairment and seizures were absent. The pedigree of the family is depicted in Figure 1A.

###  Patients’ Genotypes and Variant Description

**Figure 2:**
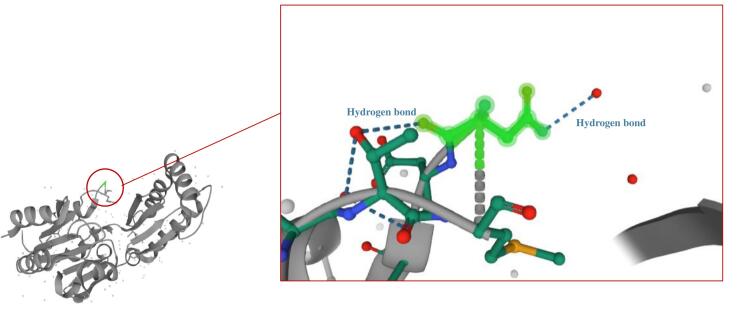
Asn216 in PMM2 Protein. Substitution of asparagine with isoleucine at position 216 likely disrupts crucial hydrogen bonds within the protein structure, as asparagine can form hydrogen bonds while isoleucine is hydrophobic

 Our study revealed compound heterozygous variants in the three affected siblings, comprising a previously reported missense mutation c.647A > T (p.N216I) and a novel splice site variant (c.640-9T > A) of uncertain significance. The electropherogram of c.640-9T > A and c.647A > T variants in the *PMM2* gene for affected individuals and normal siblings is shown in Figure 1B. *In-silico* prediction of the identified variants in the *PMM2* gene is illustrated in Table 1. The c.647A > T (p.N216I) variant was analyzed using multiple *in-silico* tools, revealing a consensus toward pathogenicity across most algorithms. MetaLR, MetaRNN, and MutPred classified the variant as pathogenic with strong confidence. REVEL and FATHMM provided moderate support for pathogenicity, with scores exceeding commonly accepted pathogenicity thresholds. CADD, with a high score of 29.5, further supports the potential deleterious nature of this variant, as values above 20 are indicative of functional impact. SIFT and LRT classified the variant as deleterious, providing additional supporting evidence. Figure 2^23^ shows Asn 216 within the protein structure. The substitution of asparagine with isoleucine at position 216 likely disrupts crucial hydrogen bonds within the protein, as asparagine possesses an amide group capable of forming hydrogen bonds, while isoleucine is hydrophobic and lacks this ability. The *in-silico* predictions for the c.640-9T > A variant suggest a high likelihood of pathogenicity. dbscSNV/SpliceAI: Classified as “Deleterious” and “Splice altering Strong,” respectively, indicating a strong potential for this variant to disrupt the splicing process. With a score of 15.77, CADD predicts this variant to be “Possibly Damaging.” While not as strong as the splicing predictions, this score still suggests a significant potential impact on gene function.

**Table 1:** *In-Silico Prediction* of Identified Variants in the PMM2 Gene

**HGVS c.**	**FATHMM**	**MetaLR**	**SIFT**	**REVEL**	**MetaRNN**	**MutPred**	**PrimateAI**	**LRT**	**Mutationtaster**	**dbscSNV/Splice AI**	**CADD**
c.640-9T > A	-	-	-	-	-	-	-	-	-	Deleterious/ splice altering strong	15.77
c.647A > T	Pathogenic moderate (-6.29)	Pathogenic strong 0.9895	Pathogenic supporting (0)	Pathogenic moderate 0.927	Pathogenic strong 0.9949	Pathogenic strong 0.972	VUS 0.5389	Pathogenic supporting (0)	VUS 1	-	29.5

## Literature Review

###  Other reports of PMM2-CDG in Iran

 According to a study by Piedade et al, no type of CDG is common in multiple countries with high rates of consanguinity, including Iran.^24^ In Iran, a Middle Eastern country with a parental consanguinity rate of approximately 40%,^25^ only three families with PMM2-CDG have been reported, including a previously reported family in 2011^26^ by our group, a family reported by Madani et al in 2021 and the present study. In 2011, a missense mutation, p.Y106F, in the *PMM2* gene was identified in a consanguineous Iranian family from the Lorestan Province with three affected children presenting with mild intellectual disability, a thin upper lip, a flat nasal bridge, and strabismus. Madani et al identified the p.G117C variant in a patient born to consanguineous parents, who presented with severe hypotonia, motor developmental delay, and elevated urinary 2-ketoglutaric acid levels.^27^

###  Mutational Spectrum among PMM2-CDG Patients 

**Figure 3:**
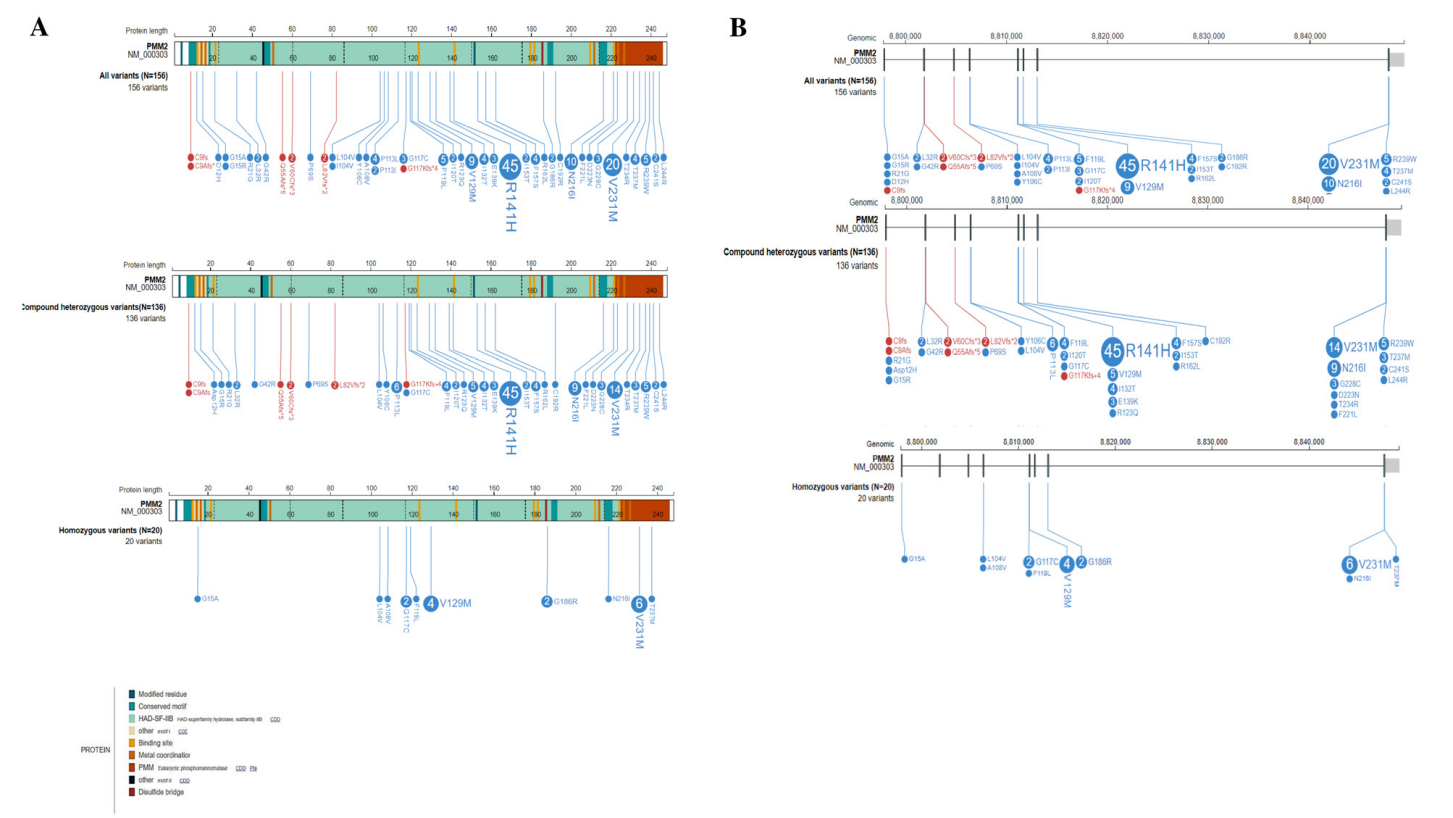
(A) *PMM2* Variants at Protein Level. (B) *PMM2 *variants at genomic level

 According to the Human Gene Mutation Database (HGMD® Professional 2023.4),^28^ approximately 158 disease-causing *PMM2* mutations have been reported so far, with a predominance of synonymous variants. We further reviewed published studies (2017‒2024) reporting *PMM2* mutations and associated clinical data. Figures 3A and 3B illustrate these variants at both the protein and genomic levels (illustrated by proteinpaint: https://proteinpaint.stjude.org/).^29^

**Figure 4:**
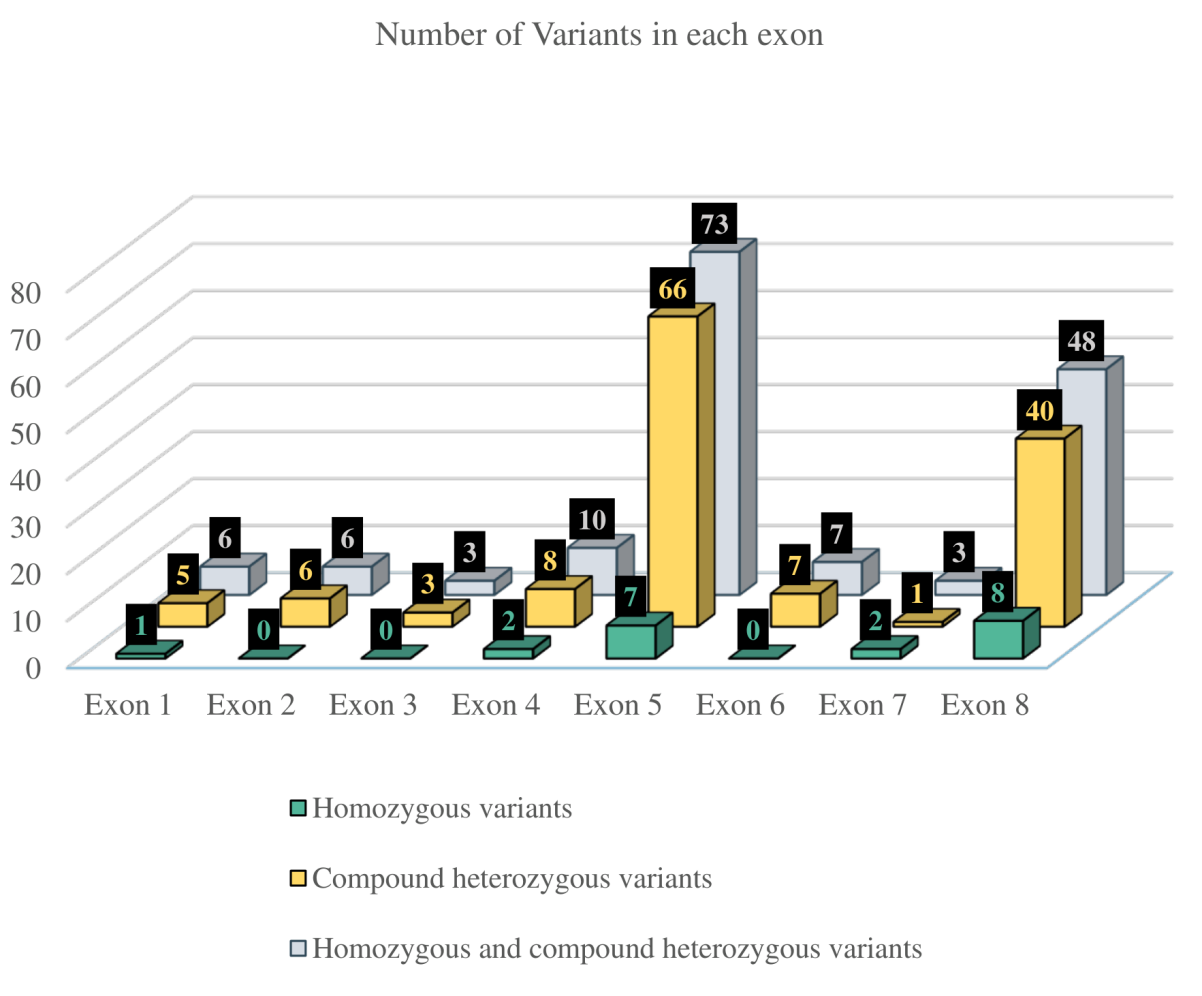
Distribution of Variants Across the Exons of the *PMM2* Gene

 Among 91 patients, 76.9% (n = 70) exhibited compound heterozygosity, while 23.1% (n = 21) demonstrated homozygosity. Further calculations were performed on a per-patient basis. Each heterozygous variant was counted once per patient (among 70 patients, 140 alleles were analyzed). For four patients, only one allele with a specified effect on protein sequence was considered. Each homozygous variant was counted once per individual. One patient carrying a homozygous variant, g.18313A > T, was excluded from further analysis due to the complex impact of the variant on the amino acid sequence of the PMM2 protein.^30^ Altogether, 156 variants were considered, including 20 (12.8%) homozygous and 136 (87.2%) compound heterozygous variants. The majority of variants were missense (94.9%, N = 148), while a smaller proportion were frameshift (5.1%, N = 8). Notably, no frameshift variants were observed among homozygous mutations. The distribution of variants across the exons of the *PMM2* gene is shown in Figure 4, revealing that recurrent variants are concentrated in exons 5 and 8, respectively.

###  Clinical Categorization of 91 Patients

 Clinical data were categorized based on the affected body systems, with the results illustrated in Table 2. Developmental delay was one of the most prevalent findings, affecting 87.9% of patients. Ocular abnormalities were common, with hypertelorism (93.3%) and strabismus (70.3%) being the most frequently observed. While a variety of skeletal and skin abnormalities were identified, their prevalence was relatively low. The most common skeletal and skin abnormality was short stature (16.4%), followed by abnormal fat pads (13.2%). Muscular system involvement was primarily characterized by hypotonia or muscle weakness, affecting 70.3% of patients. Neurological manifestations were diverse, with abnormal MRI findings being the most common. Cardiovascular abnormalities were also observed, with pericardial effusion or pericarditis being the most frequent (22%). Hepatic involvement was primarily characterized by elevated transaminases (38.5%). Finally, clotting disorders were common among PMM2-CDG patients, with antithrombin III deficiency being the most prevalent.

**Table 2:** Clinical Characterization of Previous Studies Reporting PMM2-CDG Cases With PMM2 Variants^7,27,30-56^

**Organs/clinical signs**		**Clinical Features Reported in 3 or More Cases**	**Clinical Features Reported in<3 Cases (<3.30%)**
General		Failure to thrive (27.5%)	
		Developmental delay (87.9%)	
Head & Face	Head	Microcephaly (28.6%)	Macrocephaly
	Eye	Strabismus (70.3%)	Abnormal eyebrows
		Retinitis pigmentosa (19.8%)	Abnormal eyelashes
		Nystagmus (4.4%)	Retinal dystrophy
		Hypertelorism (93.3%)	Eye light sensitivity
		Abnormal eye movement (3.3%)	Optic nerve atrophy
			Macular hypoplasia
			Decreased visual acuity
			Wrinkling of the macular retinal surface
			Cortical visual impairment
	Ear & Mouth	Hearing problem (16.5%)	Postnatal macrosomia
	Nose		Flat nasal bridge
			Prominent nares
			No reaction on nose
			Wide nasal bridge
Skeletal system	Osteoporosis/ Osteopenia (4.4%)		Kyphoscoliosis
	Scoliosis (7/9%)		Clubfoot
	Joint laxity (4.4%)		Bilateral radial aplasia
	Short stature (16.5%)		Pectus Carinatum
			Pectus excavatum
			Scapular dyskinesis (Mild winging of the scapulae)
			Talipes equinus
			Hammer toe
			Elongated slender fingers
			Spinal cord disorder
Skin	Abnormal fat distribution (13.2%)		Purpura
	Orange peel' skin (7.7%)		Petechiae
			Pressure ulcers
			pilonidal sinus,
			Skin elasticity changes
			Easy bruising
			Eczema
			Dry skin parts
Muscular system	Muscle weakness or hypotonia (70.3%)		Spasticity
	Myopathy (15.4%)		Torticollis
			Tendon Reflexes and Plantar Responses
			Spastic paraplegia
Nervous system	Ataxia (28.6%)		Non-cerebral haemorrhage
	Hyporeflexia (14.3%)		Intentional tremor
	Stroke-like episodes (14.3%)		
	Seizure or epilepsy (27.5%)		
	Stroke mimic (3.3%)		
	Cerebral thrombosis (5.5%)		
	Non-cerebral thrombosis (3.3%)		
	Cerebral haemorrhage (4.4%)		
	Abnormal MRI results (68.1%)		
	Peripheral neuropathy (16.5%)		
Urogenital system	Genital	POF or risk of POF (5.5%)	Defect in secondary sexual development
	Urinary system	Proteinuria (16.5%)	Nephrocalcinosis
		Increased renal echogenicity (15.4%)	Renal cyst/ cystic renal disorder
		Tubulopathy (5.5%)	Oliguria
		Cryptorchidism (3.3%)	Hypertension due to nephrotic syndrome
		Inguinal hernia (3.3%)	Enlarged kidney and decreased corticomedullary diameter
Cardiovascular system	Pericardial effusion/pericarditis (22%)		Conotruncal cardiac malformations
Liver problems		Hepatomegaly (18.7%)	Liver fibrosis
		Increased liver echogenicity (12.1%)	Steatosis
		Elevated transaminases (38.5%)	Liver failure
			Low haptoglobin level
Other signs	Nipple anomalies (38.5%)		Headache
	Ascites (11%)		Steatorrhea
	Edema (7.7%)		
	Behaviour changes (3.3%)		
Gastrointstinal problems	Feeding problems (22%)		Gastroesophageal reflux diseas
	Vomiting (10%)		
	Diarrhea (10%)		
Respiratory system	Pneumonia (5.5%)		Sleep apnea
	Pleural effusion (6.6%)		Episodes of cyanosis
			Sinusitis
			Pulmonary nodular amyloidosis
			Tachypnea
			Dyspnea
			Recurrent airway infections
			Hypoxemia
			Bronchopneumonia
			Respiratory distress
			Meconium aspiration syndrome
Endocrine system	Hypothyroidism (16.5%)		Thyroid binding globulin deficiency
	Hypergonadotropic hypogonadism (4.4%)		Hyperprolactinemia
	Hyperinsulinaemic hypoglycaemia (12.1%)		Panhypopituitarism/hypoplastic infundibulum
	Adrenal insufficiency (3.3%)		Hypomagnesemia
			GH deficiency
Prenatal Manifestations	Non-immune hydrops fetalis (3.3%)		Low birth weight
			Oligohydramnios
			Intrauterine growth retardation
Biochemistry	Hypocholesterolemia (4.4%)		Hypolipidaemia
	Triglyceridemia (9. 9%)		Hyperammonemia
	Hypoalbuminemia (12.1%)		Lactic acidosis
	Low serum HDL (4.4%)		Iron deficiency
			Abnormal ferritin levels
			High 2-ketoglutaric acid in urine sample
			Low circulating PCSK9 levels
			Hypoproteinemia
			Elevation of dehydrogenase
			Low microalbumin
			Low serum creatinin
			High serum creatinin
			Elevation of creatine kinase
			Low urine beta-2 microglobulin
Immunology	Hypogammaglobulinemia (3.3%)		Leucocytosis (high wbc)
Hematology	Anemia (5.5%)		Low INR
	Thrombocytopenia (5.5%)		Pancytopenia
	Thrombocytosis (3.3%)		Low factor X
	High INR (5.5%)		High factor VIII
	Prolonged PT (9.9%)		
	Factor XI deficiency (14.3%)		
	Antithrombin III deficiency (34.1%)		
	Low protein C (9.9%)		
	Low protein S (7.7%)		
	Low factor XI (9.9%)		
	Low factor IX (4.4%)		

## Discussion

 Our family harbors a previously reported missense mutation (p.N216I) and a novel intronic splice site variant (c.640-9T > A). Since 1997, multiple studies have reported the p.N216I allele in a compound heterozygous state with the p.R141H allele in Italian patients.^57,58^ The only reported homozygous genotype for this variant was observed in an unusual case of PMM2-CDG, presenting with postnatal macrosomia, distinctive bushy eyebrows with an abnormally shaped right eyebrow, and an absence of inverted nipples and fat pads. Notably, motor nerve conduction velocity in the tibialis posterior nerve of the lower limbs was normal, in contrast to other PMM2-CDG patients.^59^ As depicted in Figure 2,^23^ asparagine’s side chain contains an amide group (-CONH2), capable of forming hydrogen bonds as both a donor and acceptor. In contrast, isoleucine is a hydrophobic amino acid with a hydrocarbon side chain, unable to participate in hydrogen bonding. Thus, the substitution of asparagine with isoleucine at position 216 likely disrupts crucial protein interactions and hydrogen bonding, contributing to the observed phenotypic features in PMM2-CDG patients carrying this mutation. The other identified variant, which is a novel variant, c.640-9T > A, is located within the polypyrimidine tract of the last intron of the *PMM2* gene. To the authors’ knowledge, this variant has not been reported before, but multiple reports of intronic variant NM_000303.3:c.640-9T > G exist. A previous study identified this variant as important for the activation of a cryptic intronic splice site in fibroblast cell lines.^60^

 The PMM2-CDG is a rare disorder with only three reported families in Iran, of which two were identified by our group. The identification of recurrent variants, particularly p.R141H, p.V231M, p.N216I, and p.V129M, highlights the importance of these specific alterations in disease pathogenesis. The clustering of variants in exons 5 and 8 suggests potential mutational hotspots that may be targeted for efficient genetic testing. The absence of synonymous variants within the two conserved domains (amino acids 46‒48 and 188‒190) of the *PMM2* gene (Figure 3) may suggest that these regions are highly conserved and critical for the protein’s function. However, further studies are still needed to investigate this issue.

 As this is a descriptive study, we conducted a comprehensive review of published reports on *PMM2* variants and their associated clinical features. Common clinical findings included developmental delay, ocular problems (hypertelorism and strabismus), muscular system abnormalities (hypotonia or muscle weakness), neurological signs (abnormal MRI findings), cardiovascular system involvement (pericarditis or pericardial effusion), hepatic problems (elevated transaminases) and clotting disorders (antithrombin III deficiency). Less common findings were skeletal and skin abnormalities, and behavioral problems. This study showed that prenatal manifestations are rare among PMM2-CDG patients but they include non-immune hydrops fetalis, low birth weight, oligohydramnios and intrauterine growth retardation.

## Conclusion

 In conclusion, our study reports a novel splice variant with a nucleotide substitution in a family with PMM2-CDG and expands the knowledge on PMM2-CDG by reviewing 91 previously reported cases. The most prevalent variants and recurrent mutations occurred in exons 5 and 8 of the *PMM2* gene. A limitation of this study is that the categorization of papers was based solely on clinical signs explicitly stated by authors. This approach may have inadvertently excluded some clinical signs that, while present, were not explicitly mentioned or investigated. Furthermore, the inclusion criteria of studies reporting both genotype and phenotype data could introduce bias, as not all relevant studies may meet this specific requirement. Since the incidence of PMM2-CDG is approximately 1 in 20 000,^61^ our analysis of 91 well-documented cases—each reporting both clinical and genetic data—represents a substantial portion of the fully reported cases currently available in the literature. This allowed us to explore clinical sign classifications and identify potential genetic hotspots. However, it should be noted that not all reported cases could be included, particularly those lacking comprehensive individual-level clinical or genetic information. To mitigate these limitations and draw more robust conclusions, a comprehensive analysis of phenotypic and clinical data from a broader range of studies is necessary. For this reason, authors advocate for the creation of a comprehensive database containing both clinical and genotype data of PMM2-CDG patients.
